# Cyano-Bridged Cu-Ni Coordination Polymer Nanoflakes and Their Thermal Conversion to Mixed Cu-Ni Oxides

**DOI:** 10.3390/nano8120968

**Published:** 2018-11-23

**Authors:** Alowasheeir Azhar, Christine Young, Yusuf Valentino Kaneti, Yusuke Yamauchi, Ahmad Yacine Badjah, Mu Naushad, Mohamed Habila, Saikh Wabaidur, Zeid A. Alothman, Jeonghun Kim

**Affiliations:** 1 Key Laboratory of Eco-Chemical Engineering, College of Chemistry and Molecular Engineering, Qingdao University of Science and Technology, Qingdao 266042, China; horiatalbher@hotmail.com; 2 International Research Center for Materials Nanoarchitechtonics (WPI-MANA), National Institute for Materials Science (NIMS), 1-1 Namiki, Tsukuba, Ibaraki 305-0044, Japan; peicing@livemail.tw (C.Y.); KANETI.Valentino@nims.go.jp (Y.V.K.); 3 Faculty of Science and Engineering, Waseda University, 3-4-1 Okubo, Shinjuku, Tokyo, 169-8555, Japan; 4 School of Chemical Engineering and Australian Institute for Bioengineering and Nanotechnology (AIBN), The University of Queensland, Brisbane, QLD 4072, Australia; 5 Department of Plant & Environmental New Resources, Kyung Hee University, 1732 Deogyeong-daero, Giheung-gu, Yongin-si, Gyeonggi-do 446-701, Korea; 6 Advanced Material Research Chair, Chemistry Department P.O. Box 2455, College of Science, King Saud University (KSU), Riyadh 11451, Saudi Arabia; ybadjah@ksu.edu.sa (A.Y.B.); mnaushad@ksu.edu.sa (M.N.); mhabila@ksu.edu.sa (M.H.); tarabai22@yahoo.com.sg (S.W.); zaothman@ksu.edu.sa (Z.A.A.)

**Keywords:** coordination polymer, nanoflakes, Cu-Ni oxides, cyano-bridged, supercapacitors

## Abstract

Herein, we demonstrate the bottom-up synthesis of 2D cyano-bridged Cu-Ni coordination polymer (CP) nanoflakes through a controlled crystallization process and their conversion to Cu-Ni mixed oxides via a thermal treatment in air. The chelating effect of citrate anions effectively prevents the rapid coordination reaction between Cu^2+^ and K_2_[Ni(CN)_4_], resulting in the deceleration of the crystallization process of CPs. Specifically, with addition of trisodium citrate dehydrate, the number of nuclei formed at the early stage of the reaction is decreased. Less nuclei undergo a crystal growth by interacting with [Ni(CN)_4_]^2−^, leading to the formation of larger Cu-Ni CP nanoflakes. Following heat treatment in air, the -CN- groups present within the CP nanoflakes are removed and nanoporous Cu-Ni mixed oxide nanoflakes are generated. When tested as an electrode material for supercapacitors using a three-electrode system, the optimum Cu-Ni mixed oxide sample shows a maximum specific capacitance of 158 F g^−1^ at a current density of 1 A g^−1^. It is expected that the proposed method will be useful for the preparation of other types of 2D and 3D CPs as precursors for the creation of various nanoporous metal oxides.

## 1. Introduction

Coordination polymers (CPs) have drawn significant attention because of their recent potential for energy and environmental applications [1,2]. Nanoarchitectures constructed from various molecular building blocks can bring out new chemical and physical properties through the creation of porous frameworks. Recently, one-dimensional (1D) (e.g., nanorods, nanowires) [3], two-dimensional (2D) (e.g., nanosheets, nanoflakes) [4], three-dimensional (3D) nanostructures (e.g., nanocubes) [5] and bulk material [6] have been synthesized under various controlled synthetic conditions. Among these, 2D nanomaterials have attracted the most interest because of their novel physical or chemical properties which are distinct from their bulk counterparts [7,8,9,10]. 2D nanostructures possess highly accessible surface area which can enable guest molecules to effectively access the pore surface. Moreover, they exhibit numerous active sites which can boost the catalytic and electrochemical performance and more importantly, assembled 2D nanostructures are highly useful as membrane filters. Previously, ultrasonication-induced exfoliation methods have been used to prepare MOF (metal-organic framework) nanosheets [7,8]. Although exfoliation methods possess some important advantages, they are somewhat inconvenient due to the complicated synthetic procedures and the need for special equipment. Therefore, the development of a facile and convenient method for the large-scale preparation of 2D CPs is highly desired.

Among various CPs, cyano-bridged CPs have attracted increasing scientific interests in various fields, such as gas storage, batteries, catalysis, gas capture and separation, charge transfer, drug delivery, sensing and environmental clean-up [11,12,13,14]. Cyanide groups can act as a bridge between metals ions. The controlled thermal treatment of cyano-bridged CPs can lead to the creation of nanoporous hybrid metal oxides. During the thermal treatment of CPs in air, the metallic constituents remain in the frameworks and become oxidized to metal oxides, while the removed -CN- components can generate nanoporosity [4,5]. This method therefore can overcome the limitations of conventional methods (e.g., soft- and hard-templating methods) for the synthesis of nanoporous metals oxides [15].

In this work, we demonstrate the fabrication of a series of 2D cyano-bridged Cu-Ni CP nanoflakes through a controlled crystallization process with the assistance of trisodium citrate dihydrate (TSCD). Following thermal treatment in air, the -CN- constituents present within the Cu-Ni CP nanoflakes are removed and the metals Cu and Ni become oxidized to generate Cu-Ni mixed oxide nanoflakes with nanoporous structures. The effects of pertinent parameters, such as the concentration of TSCD and calcination temperature on the phase composition and morphology of the Cu-Ni CPs and the corresponding Cu-Ni mixed oxide nanoflakes were investigated.

Metal oxides and metal hydroxides have been studied and used for energy storage and conversion [16,17,18,19,20]. Especially, mixed metal oxides are very attractive due to the enhanced capacitance for supercapacitors [21,22,23,24,25]. Here, the electrochemical performance of the Cu-Ni mixed oxide nanoflakes calcined at different temperatures (300–500 °C) for supercapacitors was investigated using a three-electrode system and the important parameters were evaluated.

## 2. Materials and Methods

**Chemicals.** Potassium tetracyanonickelate hydrate (K_2_[Ni(CN)_4_]·xH_2_O) was purchased from FUJIFILM Wako Pure Chemical Corporation (Osaka, Japan). Copper nitrate trihydrate (Cu(NO_3_)_2_·3H_2_O) and trisodium citrate dehydrate (Na_3_C_6_H_5_O_7_.2H_2_O) were obtained from Nacalai Tesque (Japan). All chemical reagents were used as received without additional purification steps.

**Synthesis of 2D Cu-Ni CP flakes.** Cu(NO_3_)_2_·3H_2_O and trisodium citrate dehydrate (TSCD) were mixed together in 50 mL of water at room temperature to form a clear solution. In a separate bottle, K_2_[Ni(CN)_4_] was dissolved in 50 mL water to form another clear solution. Then, the two solutions were mixed under magnetic stirring until the solution became clear and the resulting mixture was aged at room temperature for 24 h. After completion of the reaction, the precipitate was collected by centrifugation. Then, the product was thoroughly washed with water and ethanol for several times. Following drying at ambient temperature, 2D Cu-Ni CP nanoflakes were obtained. The amount of trisodium citrate dehydrate (TSCD) was varied to prepare Cu-Ni nanoflakes with different size. The sample names are abbreviated as Cu-Ni*x* where *x* is the amount of TSCD (g). The detailed quantity of chemicals used for the synthesis is summarized in Table 1.

**Thermal conversion from 2D Cu-Ni nanoflakes to mixed metal oxides.** The obtained Cu-Ni_0.20 nanoflakes were used as the precursor and heated from room temperature to the desired temperature with a heating rate of 5 °C min^−1^ in air. After reaching the targeted temperature (300, 400 and 500 °C), the samples were annealed for 1 h to complete the thermal decomposition of the Cu-Ni CP nanoflakes and then, they were cooled naturally to room temperature. The samples are labeled as Cu-Ni_*x*_*y* where *x* is the amount of TSCD (g) and *y* is the applied calcination temperature.

**Characterization.** Wide-angle XRD patterns of the samples were collected using a Rigaku RINT 2500X diffractometer with monochromated Cu-K*α* radiation (40 kV, 40 mA) at a scanning rate of 1° min^−1^. The parallel beam optics was used, which is the general way to analyze the powder sample. The morphological characterization of the samples was performed using a Hitachi SU8000 scanning electron microscope (SEM) operated at an accelerating voltage of 5 kV. Transmission electron microscopy (TEM) observation was performed using a JEM-2100F TEM system that was operated at 200 kV and equipped with energy-dispersive spectrometer (EDS). X-ray photoelectron spectroscopy (XPS) measurements of the samples were conducted using a PHI Quantera SXM (ULVAC–PHI with CasaXPS software) instrument which employed an Al-K*α* X-ray source. The take-off angle was 45 degree. The fitting was done with linear fitting. All binding energies were calibrated with reference to C1s (285.0 eV). The crystal structure of the compound after calcination at 500 °C was obtained by the Pawley method using two starting structure model of CuO and NiO, with the GSAS II program and plotted with zero-shift correction and background subtraction [26]. Thermogravimetric-differential thermal analysis (TG-DTA) of the samples was performed using a Hitachi HT–Seiko Exter 6300 TG/DTA from room temperature to 1000 °C under air at a fixed heating rate of 5 °C min^–1^. N_2_ adsorption-desorption isotherms of the samples were achieved by employing a Quantachrome Autosorb gas sorption system at 77 K. Fourier transform infrared spectroscopy (FTIR) was used to identify the chemical constituents present on the samples. The samples were mixed with potassium bromide (KBr) and pressed into pellets. The FTIR spectra were collected at room temperature by using the Thermoscientific Nicolet 4700 instrument. UV-vis spectra were collected with the use of V-570 UV-Vis-NIR spectrophotometer made by JASCO.

**Electrochemical measurements.** The electrochemical measurements were performed by using an electrochemical workstation (CHI 660e, CH Instruments). For the three-electrode measurements, Ag/AgCl and platinum wire were utilized as the reference electrode and counter electrode, respectively. The glassy carbon substrate (1 cm^2^) was used as the current collector. The working electrode was prepared by coating a slurry containing the active material (1 mg, 85 wt.%), super P (10 wt.%), polyvinylidene fluoride binder (PVDF) (5 wt.%), and N-methyl-2-pyrrolidone on carbon paper as the current collector. The proper amount of slurry was carefully dropped on the glassy carbon electrode (GCE). The coated electrode was dried in a vacuum oven at 80 °C for 12 h. All the electrochemical measurements were conducted using 6 M KOH (aq) as the electrolyte. The gravimetric capacitances were calculated from the CV curves by using the following equation:

$$C_{g}=\frac{1}{ms\left(V_{f}-V_{i}\right)}\int_{V_{i}}^{V_{f}}I\left(V\right)dV$$

where ‘*C*_g_’ is gravimetric capacitance (F g^−1^), ‘*s*’ is the potential scan rate, ‘*V*’ is potential window, ‘*I*’ is current (A), ‘*t*’ is discharge time (s) and ‘*m*’ is the mass of active material in gram.

The galvanostatic charge-discharge (GCD) measurements were carried out at varying current densities of 1, 2, 4, 6 and 10 A g^−1^. The gravimetric capacitances were calculated from the GCD curves via the following equation:

$$C_{g}=\frac{I\times \int V\;dt}{M\times \Delta V^{2}}$$

where ‘*C*_g_’ represents the gravimetric capacitance (F g^−1^), ‘Δ*V*’ represents the potential window, ‘*I*’ represents the current (A), ‘*t*’ represents the discharge time (s), and ‘*M*’ represents the total mass of active material (g).

## 3. Results

**Figure 1:**
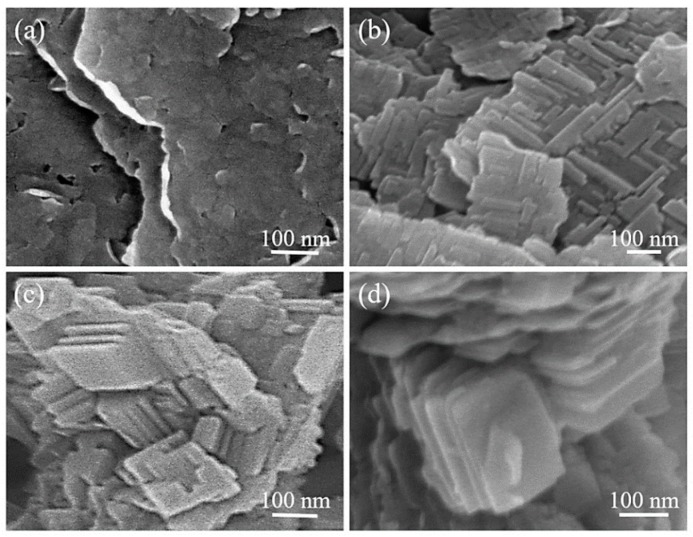
SEM images of 2D Cu-Ni CPs prepared with different amount of TSCD: (**a**) Cu-Ni_0.00; (**b**) Cu-Ni_0.15; (**c**) Cu-Ni_0.20 and (**d**) Cu-Ni_0.25).

The morphology of the as-prepared Cu-Ni CP particles was characterized using SEM, as presented in Figure 1. It is clear from this figure that the concentration of TSCD is critical for controlling the structure and size of the formed 2D CP nanoflakes. Without TSCD, only irregularly- shaped nanoparticles with severe aggregation are obtained. (Figure 1a). In contrast, with increasing amount of the chelating agent, the nanoflake morphology becomes more obvious. It is well known that the particle size of nanoparticles is strongly influenced by the balance between nucleation and crystal growth. Based on the UV-vis spectra (Figure S1), it can be observed that following the addition of TSCD, the intensity of the maxima absorption peak of the Cu(NO_3_)_2_ solution is greatly enhanced and the position of the peak is slightly shifted. These changes are largely caused by the coordination reaction between citrate ions and Cu^2+^ ions [27,28]. The addition of TSCD creates a chelating effect which decelerates the coordination reaction between Cu^2+^ and K_2_[Ni(CN)_4_], thus leading to the reduction in crystallization speed of the CP particles. Our group has previously discovered that in the absence of citrate ions, very rapid formation of CP was achieved, and the reaction was terminated within a relatively short time. Thus, by implementing TSCD into the reaction system, the crystallization process of CP was delayed [29,30]. Our previous ^1^H NMR study demonstrated that that citrate anions can stabilize the metal ions (e.g., Ni^2+^) in the solution and the free metal ions released from the citrate complex can slowly react with K_2_[Ni(CN)]_4_ to form the cyano-bridged Ni-Ni CP [4]. In the current reaction system, Cu^2+^ ions are freed in a controlled manner from the Cu-citrate complex and react with [Ni(CN)_4_]^2−^ at the beginning of the reaction. This reaction leads to the generation of nuclei which undergo further growth by interacting with the free Cu^2+^ ions and [Ni(CN)_4_]^2−^ to form the final Cu-Ni CP. Therefore, with increasing concentration of TSCD, the amount of nuclei generated at the beginning of the reaction is reduced (Figure 1c,d). As a result, fewer nuclei undergo crystal growth by interacting with [Ni(CN)_4_]^2−^ and Cu-Ni CP nanoflakes with larger sizes are obtained. In contrast, at lower concentrations of TSCD (Figure 1a,b), more free Cu^2+^ ions are available to react with [Ni(CN)_4_]^2−^ immediately. Consequently, there is a greater amount of nuclei which undergo rapid growth at the initial stage of the reaction, leading to smaller-sized nanoflakes, as shown in Figure 1a. TEM images of the Cu-Ni CP synthesized under the typical conditions (Cu-Ni_0.20) are shown in Figure S2. The flake-like morphology is clearly observed over the entire area.

**Figure 2:**
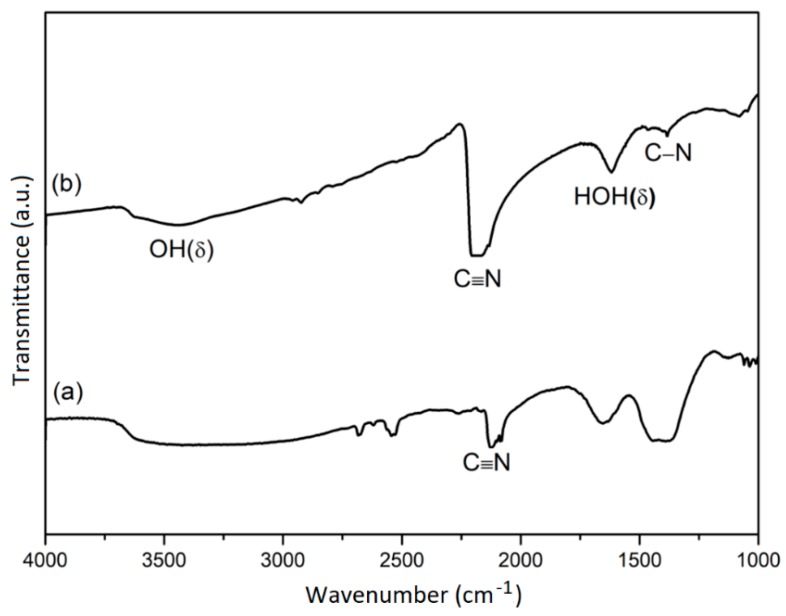
FTIR spectra of (**a**) K_2_Ni(CN)_4_·H_2_O and (**b**) Cu-Ni_0.20.

Figure 2 shows the FTIR spectrum of Cu-Ni_0.20, in which the presence of cyano-bridged complexes is identified by the existence of a sharp stretching band (CN) at 2000-2200 cm^−1^ [31,32,33]. The FTIR spectrum of the K_2_[Ni(CN)_4_]·*x*H_2_O shows a sharp stretching vibration (CN) at 2123 cm^–1^ [34]. In the FTIR spectrum of Cu-Ni_0.20, there is a shift of the stretching vibration (CN) band to a higher wavenumber of 2170 cm^−1^ [35,36]. This shift is caused by the stringing of the CN bond with other metal ions (Cu-CN-Ni) due to the kinematic effect [36,37]. In addition, the two peaks at 3450 cm^−1^ and 1616 cm^−1^ correspond to the O-H stretching vibration and the H-O-H bending vibration of water existing in the Cu-Ni_0.20 sample [31,32,38,39].

In order to investigate the porosity, nitrogen (N_2_) adsorption-desorption measurements were performed for all the CPs prepared with different amount of TSCD. Prior to the measurements, all the samples were degassed at 150 °C for 24 h. This degassing condition is sufficient for achieving complete removal of water molecules from the Cu-Ni CPs, as will be explained in the later section. The BET surface areas of the Cu-Ni_0.15 and Cu-Ni_0.20 samples are 49.0 and 47.7 m^2^ g^−1^, respectively, while the surface area of the Cu-Ni sample prepared without TSCD (Cu-Ni_0.00) is the lowest at 31.5 m^2^ g^−1^. In the case of irregularly shaped particles (Cu-Ni_0.00), N_2_ gas cannot easily access the undeveloped pores, thereby leading to a low surface area. By further increasing the amount of TSCD, the surface area of Cu-Ni_0.25 is decreased to 34.4 m^2^ g^−1^. Thus, the accessibility of N_2_ gas to the particle interior varies depending on particle sizes.

The TG-DTA analysis of the typical sample Cu-Ni_0.2 under air atmosphere is shown in Figure S3. A small weight loss at temperatures below 200 °C is attributed to the release of water molecules existing in the Cu-Ni CPs. Following this, a clear endothermic reaction occurs at around 350 °C and a large weight loss is observed at this stage as -CN- constituents are removed, and the metallic constituents are oxidized in air. In this work, we calcined the CPs at 300 °C (minimal), however after reaching the designated temperature, the samples were annealed for 1 h to complete the thermal decomposition of Cu-Ni flakes. This thermal treatment is sufficient to completely remove the -CN- groups present within the CP nanoflakes and no carbon is present in the final product.

**Figure 3:**
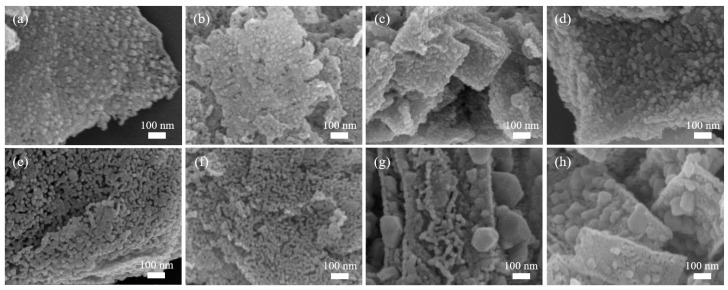
SEM images of (**a**) Cu-Ni_0.00_300; (**b**) Cu-Ni_0.15_300; (**c**) Cu-Ni_0.20_300; (**d**) Cu-Ni_0.25_300; (**e**) Cu-Ni_0.00_500; (**f**) Cu-Ni_0.15_500; (**g**) Cu-Ni_0.20_500 and (**h**) Cu-Ni_0.25_500.

**Figure 4:**
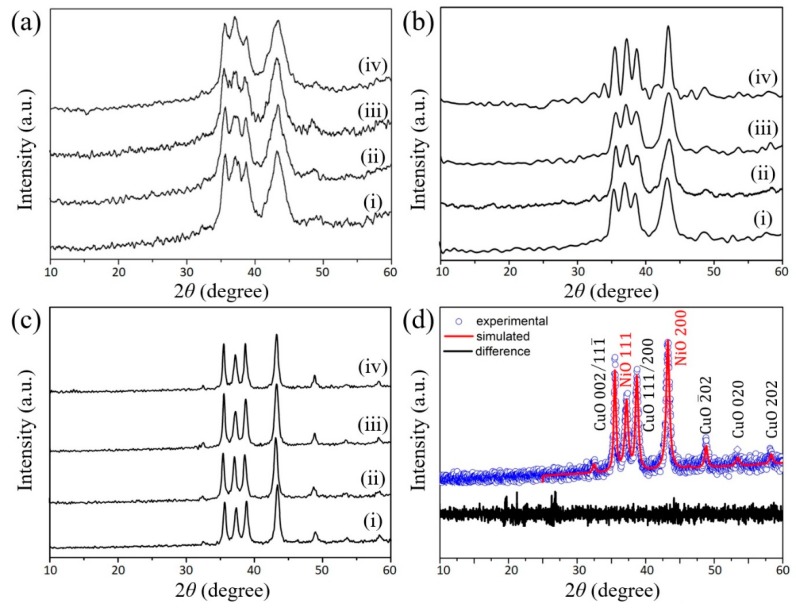
Wide-angle XRD patterns of the calcined samples at various temperatures ((**a**) 300 °C; (**b**) 400 °C and (**c**) 500 °C) of (**i**) Cu-Ni_0.00, (**ii**) Cu-Ni_0.15, (**iii**) Cu-Ni_0.20 and (**iv**) Cu-Ni_0.25; (**d**) Powder XRD pattern of Cu-Ni_0.20_500 and refinement by the Pawley method.

The SEM images of the various nanoporous metal oxide samples derived from the calcination of Cu-Ni_0.2 CP nanoflakes at different temperatures (300 and 500 °C) are shown in Figure 3. The sample calcined at 300 °C almost entirely retains the original morphology of the Cu-Ni CP before calcination. However, when the applied calcination temperature is increased, a large structural change occurs through the fusion of several pores/voids via further crystallization of the framework. Wide-angle XRD patterns for the various calcined samples are shown in Figure 4a–c. The XRD patterns of the samples calcined at 300, 400 and 500 °C show no peaks originating from impurities or unoxidized Cu or Ni phase. All the peaks are in agreement with the standard JCPDS cards for CuO (No. 48-1548) and NiO (No. 47-1049). With the increase of applied calcination temperature, the full width at half maximums (FWHMs) are decreased, suggesting that the average crystallite sizes increase. Elemental analysis for the sample calcined at 300 °C (Cu-Ni_0.20_300) shows that the resulting metal oxide product has similar content of Cu (37.90 at.%) and Ni (37.20 at.%). This ratio is almost similar to the starting composition before calcination, indicating the absence of evaporation of the metallic constituents.

**Figure 5:**
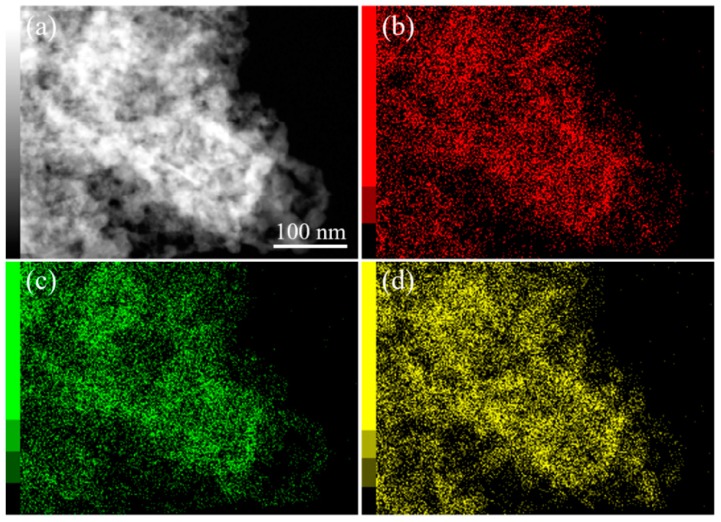
(**a**) HAADF-STEM image and (**b**–**d**) the corresponding elemental mapping ((**b**) oxygen, (**c**) copper, and (**d**) nickel) images of Cu-Ni_0.20_300.

After thermal treatment at 300 °C, the sample (Cu-Ni_0.20_300) was characterized by TEM (Figure S4). Small crystals with sizes between 5–10 nm aggregate together to form the nanoporous architecture. From the HRTEM image of this sample, clear lattice fringes with respective *d*-spacing of 0.21 nm and 0.24 nm are observed, which can be indexed to the (111) and (200) planes of NiO crystal, while the *d*-spacing of 0.25 nm is well matched with the *d*-spacing of (111) plane of CuO [40,41]. High angle annular dark field scanning transmission electron microscope (HAADF-STEM) images and the corresponding elemental mapping data confirm the flake-like structure and reveal the uniform distribution of the composing elements, Ni, Cu and O throughout the entire area (Figure 5). These results therefore confirm the successful conversion of the Cu-Ni CPs into nanoporous oxides after calcination.

The surface area of the sample calcined at 300 °C (Cu-Ni_0.20_300) is higher than the other samples calcined at higher temperatures (Cu-Ni_0.20_400 and Cu-Ni_0.20_500). As the applied thermal temperature is increased, the surface area is greatly decreased from 43.1 m^2^ g^−1^ (Cu-Ni_0.20_300) to 11.1 m^2^ g^−1^ (Cu-Ni_0.20_400) and 6.2 m^2^ g^−1^ (Cu-Ni_0.20_500) due to the fusion of pores during the crystallization of the framework. Furthermore, with increasing calcination temperature, larger crystals are observed on the surface of the samples (Figure 3g,h).

Figure 4d shows the XRD pattern of Cu-Ni_0.20_500 and refinement by the Pawley method. Clearly, the presence of two phases (CuO and NiO) are confirmed. The space group of *Fm*-3*m* of NiO structure with lattice constants, *a* = 4.178(1) Å, *b* = 4.178(1) Å and *c* = 4.178(1) Å as well as CuO structure with *C*2/*c* space group with lattice parameters, *a* = 4.683(7) Å, *b* = 3.422(6) Å and *c* = 5.128(8) Å, respectively. Finally, the reliability factors are identified as *R*_wp_=29.25% and *R*_B_=12.32% and GOF= 1.04. The results of the structural and crystallographic analyses of the sample Cu-Ni_0.20_500 are summarized in Table 2.

**Figure 6:**
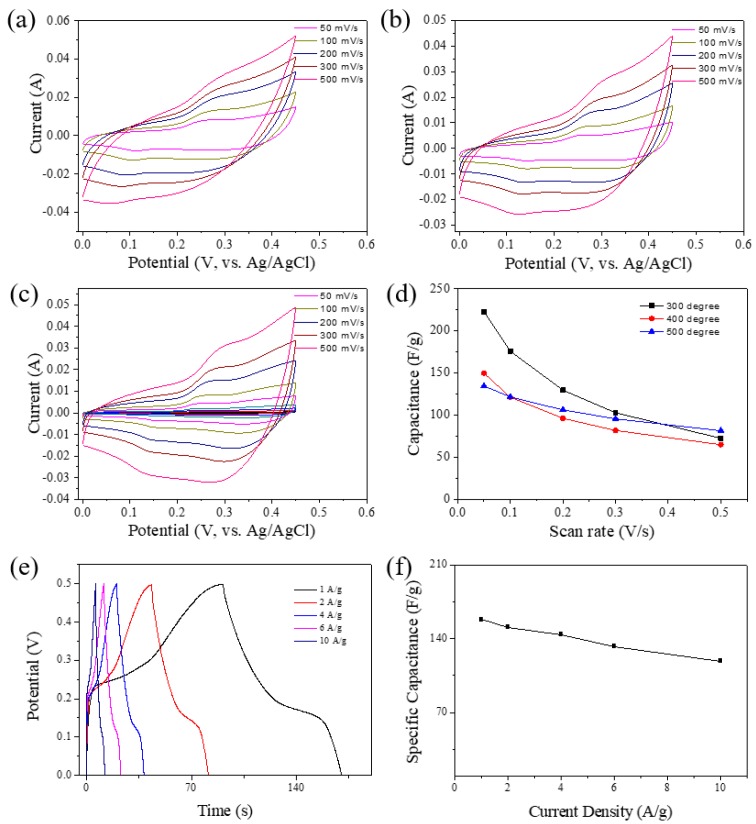
CV curves of Cu-Ni oxides calcined at (**a**) 300 °C, (**b**) 400 °C, and (**c**) 500 °C at various scan rates ((**a**) Cu-Ni_0.20_300, (**b**) Cu-Ni_0.20_400, and (**c**) Cu-Ni_0.20_500). (**d**) Specific capacitance versus scan rate plots for all the samples. (**e**) GCD curves and (**f**) specific capacitance of Cu-Ni_0.20_300 at different current densities.

It is well-known that transition metal oxides are highly useful for supercapacitor applications due to their redox activity and high specific capacitance [41,42]. To evaluate the electrochemical storage performance of all the Cu-Ni oxide samples, a three-electrode system was used with 6 M KOH as the electrolyte. Cyclic voltammetry (CV) measurements of Cu-Ni_0.20_300, Cu-Ni_0.20_400 and Cu-Ni_0.20_500 were conducted in the potential window of 0–0.5 V which is the well-known potential window of Cu-Ni oxide for supercapacitor application (Figure 6a–c) [41]. Based on the CV curves, the specific capacitance values of Cu-Ni_0.20_300, Cu-Ni_0.20_400 and Cu-Ni_0.20_500 at a scan rate of 50 mV s^−1^ are determined to be 222.6, 149.6, and 134.5 F g^−1^, respectively (Figure 6d). Among all the samples, Cu-Ni_0.20_300 shows the highest specific capacitance at all scan rates because of its high surface area, although it shows lower capacitance retention at higher scan rates. The sample Cu-Ni_0.20_500 exhibits good capacitance retention of 71%, while the sample Cu-Ni_0.20_300 has poor capacitance retention of 46%. This may be attributed to the higher stability of Cu-Ni oxide composite calcined at higher temperatures. In addition, galvanostatic charge-discharge (GCD) measurements were also carried out for Cu-Ni_0.20_300 at different current densities. This sample exhibits a specific capacitance of 158 F g^−1^ at a current density of 1 A g^−1^ and displays good stability up to 10 A g^−1^ (Figure 6e,f).

## 4. Conclusions

We have demonstrated the successful fabrication of 2D Cu-Ni CP nanoflakes via a controlled crystallization process with the assistance of TSCD. It is found that the concentration of TSCD strongly influenced the size and morphology of the resulting Cu-Ni CPs, with higher concentration of TSCD leading to more well-defined and larger-sized nanoflakes due to the reduction in crystallization speed of Cu-Ni CPs. These Cu-Ni CPs were subsequently converted into nanoporous Cu-Ni mixed oxides via thermal treatment in air at 300–500 °C and they showed respectable electrochemical performance for supercapacitors with a maximum specific capacitance 158 F g^−1^ at 1 A g^−1^ and good capacitance retention of 71%. It is expected that the proposed method will be useful for the preparation of other types of 2D and 3D CPs as precursors for the synthesis of various nanoporous metal oxides for energy and environmental applications.

## Figures and Tables

**Table 1:** Synthetic conditions of the various 2D Cu-Ni CPs.

Sample Names	Cu (NO_3_)_2_·3H_2_O (g)	K_2_[Ni(CN)_4_] (g)	TSCD (g)	Surface Area (m^2^ g^−1^)
Cu-Ni_0.00	0.120	0.120	0.00	31.54
Cu-Ni_0.15	0.120	0.120	0.15	48.97
Cu-Ni_0.20	0.120	0.120	0.20	47.69
Cu-Ni_0.25	0.120	0.120	0.25	34.43

**Table 2:** Crystallographic data for the compound Cu–Ni_0.20_500, obtained by refinement by the Pawley method.

Compound	1	2
**Formula**	CuO	NiO
**Space group**	*C*2/*c*	*Fm* $\bar{3}$ *m*
**a/Å**	4.683(7)	4.178(1)
**b/Å**	3.422(6)	4.178(1)
**c/Å**	5.128(8)	4.178(1)
**α(^o^)**	90.00	90.00
**β(^o^)**	99.54	90.00
**γ(^o^)**	90.00	90.00
**V/Å^3^**	81.0798	72.9298
*w*R(%) *	3.2	1.6

* *w*R is weighted reliability factors with background.

## Supplementary Materials

The following are available online at http://www.mdpi.com/2079-4991/8/12/968/s1, Figure S1: UV-vis spectra of Cu(NO_3_)_2_ solution with and without TSCD, Figure S2: TEM image of Cu-Ni_0.2, Figure S3: TG-DTA data of Cu–Ni_0.2. The measurement was carried out in air, Figure S4: TEM image of Cu-Ni_0.2_300. Figure S5: High-resolution XPS spectra for (a) Cu 2p, (b) Ni 2p and (c) O 1s of Cu-Ni_0.20_300.
