# Supplementary material for: Cyano-Bridged Cu-Ni Coordination Polymer Nanoflakes and Their Thermal Conversion to Mixed Cu-Ni Oxides
Source: Nanomaterials (Basel). 2018 Nov 23;8(12):968. doi: 10.3390/nano8120968 (PMC6315628; doi:10.3390/nano8120968)
Supplement: Supplementary file 1 [file nanomaterials-08-00968-s001.pdf]

Cyano-Bridged Cu-Ni Coordination Polymer Nanoflakes and Their Thermal Conversion to Mixed Cu-Ni Oxides

Alowasheeir Azhar [1,2,3], Christine Young [2], Yusuf Valentino Kaneti [2], Yusuke Yamauchi [1,4,5,*], Ahmad Yacine Badjah [6], Mu Naushad [6], Mohamed Habila [6], Saikh Wabaidur [6], Zeid A. Alothman [6] and Jeonghun Kim [1,4,*]

[1] Key Laboratory of Eco-chemical Engineering, College of Chemistry and Molecular Engineering, College of Chemistry and Molecular Engineering, Qingdao University of Science and Technology, Qingdao 266042, China; horiatalbher@hotmail.com (A.A.)

[2] International Research Center for Materials Nanoarchitechtonics (WPI-MANA), National Institute for Materials Science (NIMS), 1-1 Namiki, Tsukuba, Ibaraki 305-0044, Japan;
peicing@livemail.tw (C.Y.); KANETI.Valentino@nims.go.jp (Y.V.K.)

[3] Faculty of Science and Engineering, Waseda University, 3-4-1 Okubo, Shinjuku, Tokyo, 169-8555, Japan;

[4] School of Chemical Engineering and Australian Institute for Bioengineering and Nanotechnology (AIBN), The University of Queensland, Brisbane, QLD 4072, Australia

[5] Department of Plant & Environmental New Resources, Kyung Hee University, 1732 Deogyeong-daero, Giheung-gu, Yongin-si, Gyeonggi-do 446-701, Korea

[6] Advanced Material Research Chair, Chemistry Department P. O. Box 2455, College of Science, King Saud University (KSU), Riyadh 11451, Saudi Arabia; ybadjah@ksu.edu.sa (A.Y.B.); mnaushad@ksu.edu.sa (M.N.); mhabila@ksu.edu.sa (M.H.); tarabai22@yahoo.com.sg (S.W.); zaothman@ksu.edu.sa (Z.A.A.)

* Correspondence: y.yamauchi@uq.edu.au (Y.Y.); jeonghun.kim@uq.edu.au (J.K.)

Received: 1 October 2018; Accepted: 14 November 2018; Published: 23 November 2018

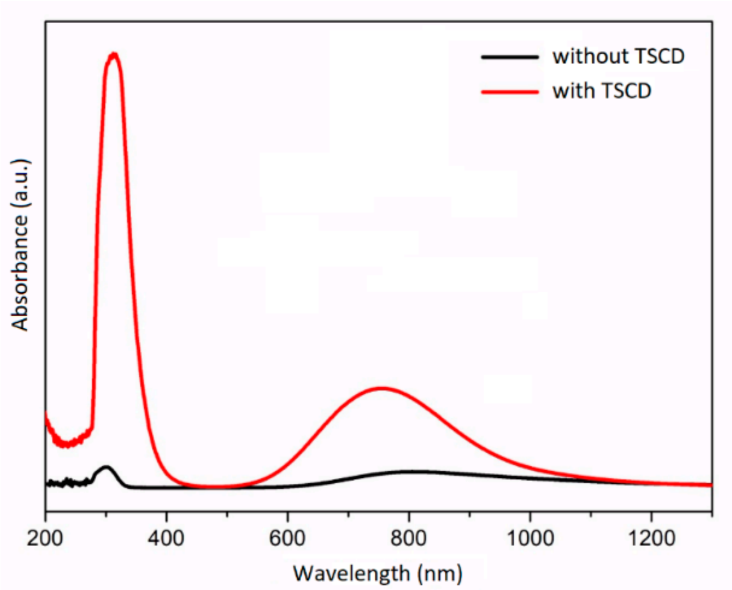

Figure S1. UV-vis spectra of $Cu(NO_3)_2$ solution with and without TSCD.

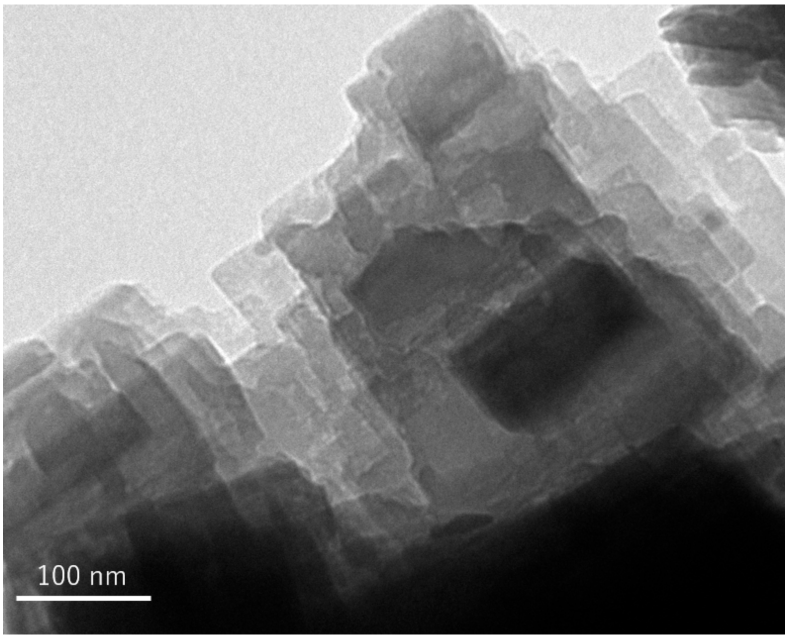

Figure S2. TEM image of Cu-Ni_0.2.

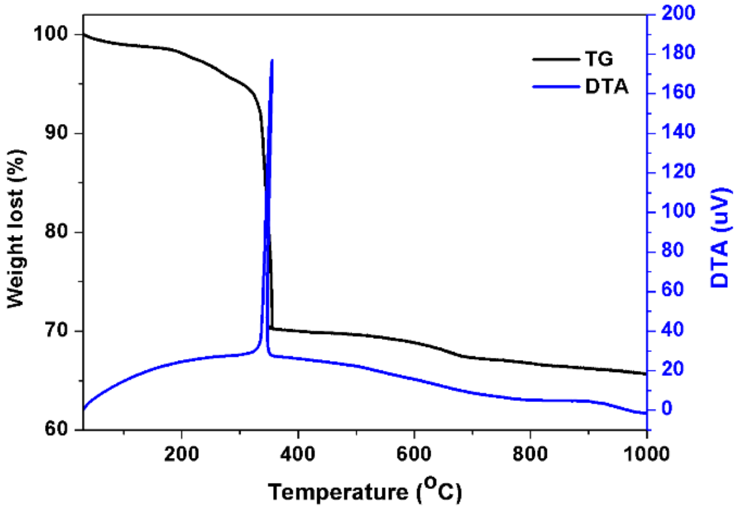

Figure S3. TG-DTA data of Cu–Ni_0.2. The measurement was carried out in air.

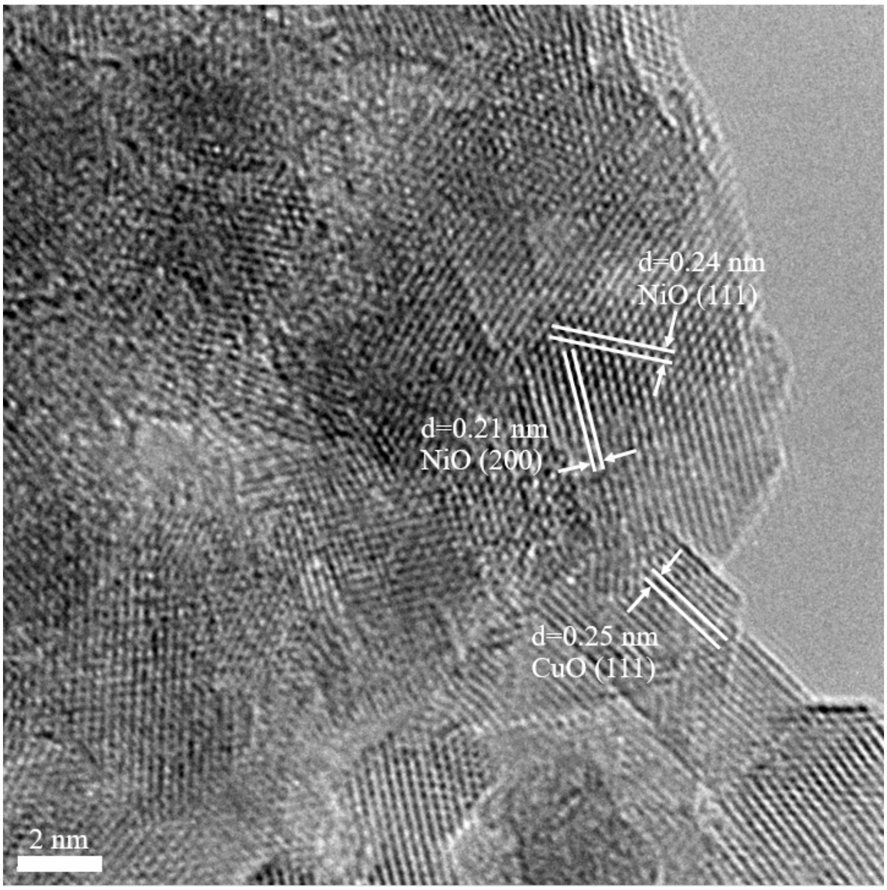

Figure S4. TEM image of Cu-Ni_0.2_300.

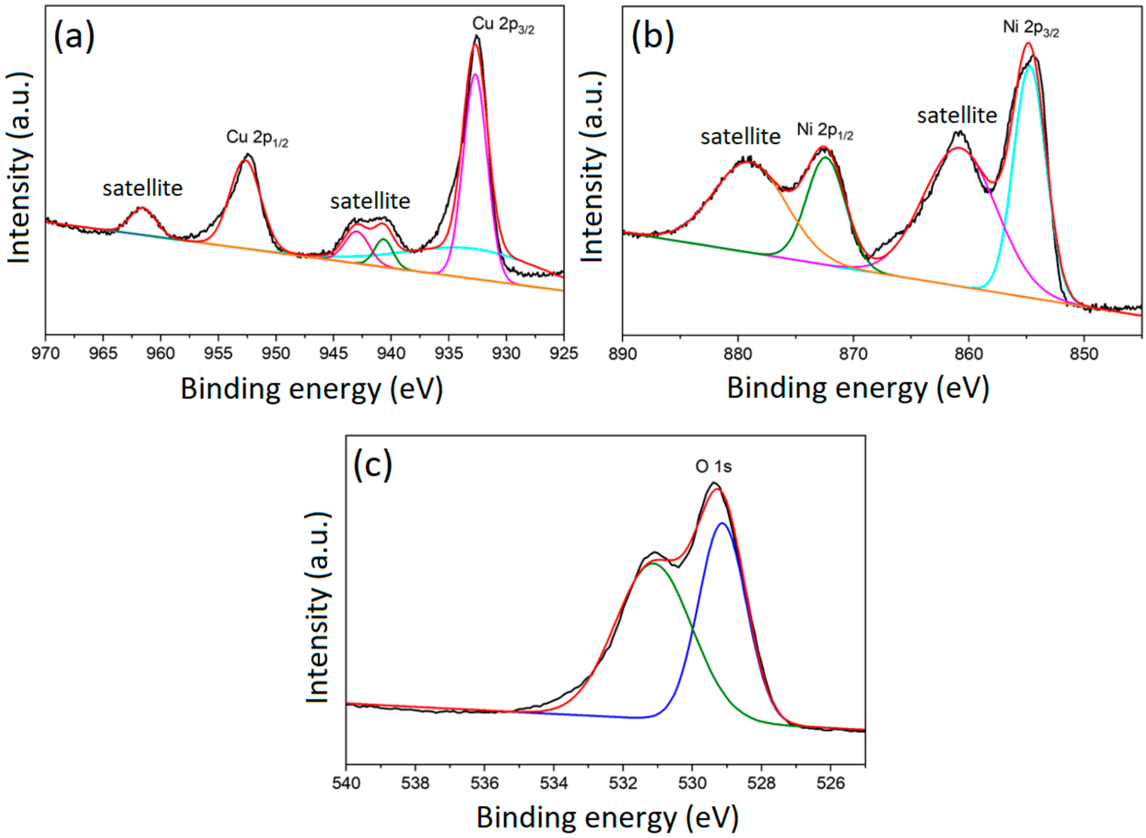

Figure S5. High-resolution XPS spectra for (a) Cu 2p, (b) Ni 2p and (c) O 1s of Cu-Ni_0.20_300.
